# *In situ*/*operando* method for energy stability measurement of synchrotron radiation

**DOI:** 10.1107/S160057752400852X

**Published:** 2024-10-15

**Authors:** Shangyu Si, Zhongliang Li, Lian Xue, Ke Li

**Affiliations:** a Shanghai Synchrotron Radiation Facility Shanghai Advanced Research Institute, Chinese Academy of Sciences 239 Zhangheng Road, Pudong New District Pudong201204 People’s Republic of China; Tohoku University, Japan

**Keywords:** synchrotron radiation, energy stability, *in situ*, Laue diffraction

## Abstract

A novel *in situ*/*operando* method is introduced to measure the photon beam stability of synchrotron radiation based on orthogonal diffraction imaging of a Laue crystal/analyzer, which can decouple the energy/wavelength and Bragg angle of the photon beam using the dispersion effect in the diffraction process. The method was used to measure the energy jitter and drift of the photon beam on BL09B and BL16U at the Shanghai Synchrotron Radiation Facility. The experimental results show that this method can provide a fast way to measure the beam stability of different light sources including bending magnet and undulator with meV-level energy resolution and ms-level time response.

## Abstract 2

A novel *in situ*/*operando* method is introduced to measure the photon beam stability of synchrotron radiation, which can provide a fast way to measure the beam stability of different light sources including bending magnet and undulator with meV-level energy resolution and ms-level time response.

## 1. Introduction

Synchrotron radiation (SR) is an X-ray light source with excellent characteristics of high brightness, wide spectrum, good polarization and coherence, providing a variety of experimental techniques for scientific research (Willmott, 2011 ▸). In the past decade, a new generation of SR facilities have sparked an exciting revolution in X-ray science based on ultra-high brightness and spatiotemporal resolution (Huang *et al.*, 2021 ▸), which put forward higher requirements for *in situ* measurements of the beamline. The stability of the photon beam is one of the critical SR parameters as it is an essential consideration for experimental design, data acquisition and analysis (Moretti Sala *et al.*, 2018 ▸; Si *et al.*, 2024 ▸). Most of the available photon beam monitors are based on direct or indirect measurement of the total flux (Aoyagi *et al.*, 2001 ▸; Nida *et al.*, 2019 ▸); since general imaging detectors lack the ability to resolve the energy distribution, the energy distribution information of the diffracted beam cannot be obtained and the monitors are sensitive to the beam position only, having no energy resolution.

In this paper, we introduce a novel *in situ*/*operando* method for measuring the photon beam stability with meV-level energy resolution based on orthogonal diffraction imaging (Si *et al.*, 2023 ▸) of a Laue crystal/analyzer which can decouple the energy/wavelength and Bragg angle of the photon beam using the dispersion effect in the diffraction process. The energy distribution of the diffracted beam is then converted into an intensity distribution that can be directly detected by an imaging detector. An sCMOS camera was placed downstream of the orthogonal analyzer to record the divided diffracted and transmitted beam spots of different times. Significant advantages of *in situ*/*operando* approaches over *ex situ* characterization are as follows: (1) *in situ* measurements can provide better reliability and higher precision for the data analysis; and (2) *in operando* measurements continuously monitor the photon beam under operating conditions and provide information closer to real-time operation.

## 2. Optical layout

**Figure 1:**
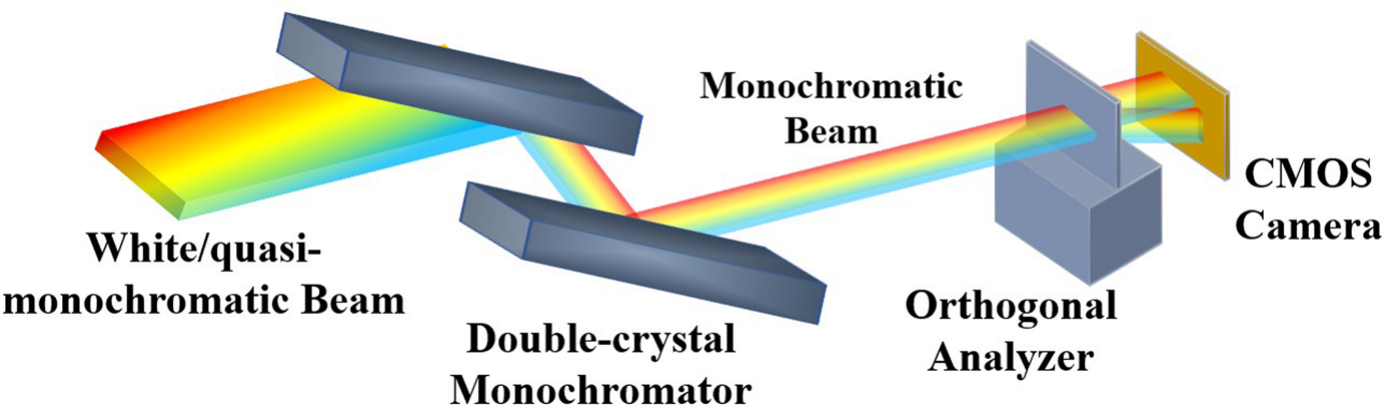
Optical layout of the beam energy stability measurement system.

A schematic view of the measurement system is shown in Fig. 1 ▸. White/quasi-monochromatic beam is diffracted by the Si(111) lattice planes of a double-crystal monochromator (DCM) in non-dispersion configuration (+*n*, −*n*), resulting in a monochromatic beam. According to Bragg’s law, 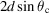 = 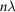, diffraction couples the photon wavelength/energy to the Bragg angle on the lattice planes within the crystals of the DCM, and the diffracted beam will contain a vertical spread of energies due to the dispersion effect. The diffraction plane index of the analyzer crystal is Si(111); the plane direction of that is perpendicular to the DCM and can decouple the energy/wavelength and angle based on the dispersion effect in the horizontal direction (Si *et al.*, 2023 ▸). Combined with a beam splitting technique based on symmetrical Laue diffraction (Zhao *et al.*, 2022 ▸), an *in situ*/*operando* measurement can be achieved, and the energy stability of the beam can be monitored in real time without affecting the downstream user experiment. A high-speed sCMOS camera was placed approximately 0.2 m downstream from the orthogonal analyzer to record splitting beam spots at different times. The pixel size of the detector was 6.5 µm, the effective area was about 13 mm × 13 mm, and the shortest exposure time was 1 ms. The energy resolution of the system can be expressed as 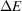 = 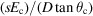 in which *D* is the distance from the light source to the detector, *s* is the pixel size of the detector, and *E*_c_ and θ_c_ are the Bragg energy and angle, respectively.

**Figure 2:**
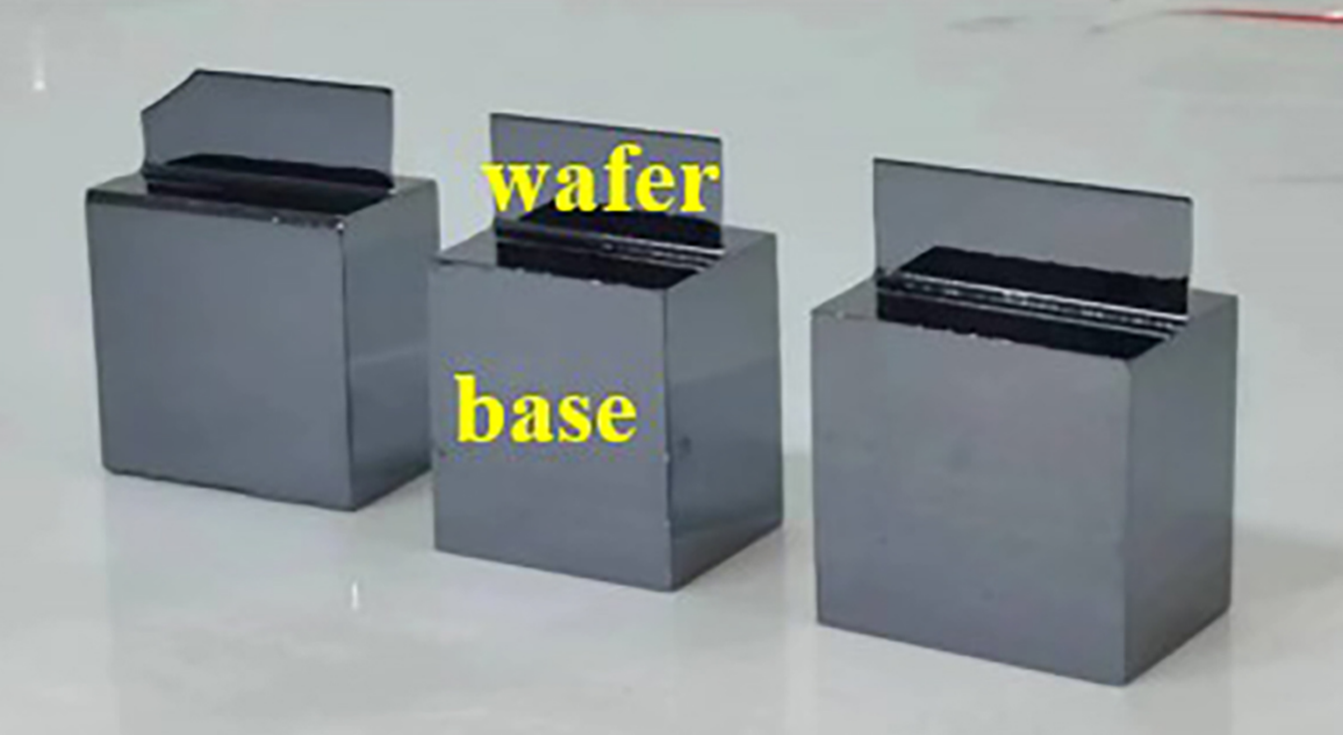
Photograph of the orthogonal analyzer.

The analyzer crystal is in a spatial orthogonal configuration with the DCM – the orthogonal analyzer is the key component in the measurement system. As shown in Fig. 2 ▸, the orthogonal analyzer is machined from a single piece of perfect monocrystalline silicon, which includes a base and a ‘thin’ diffracted wafer. The function of the base is to isolate the stress caused by the clamping process, and the wafer goes through a special corrosion and polishing process to minimize lattice deformation caused by the machining process. The size of the analyzer base used in the experiment is 40 mm (L) × 30 mm (W) × 40 mm (H) and the thickness of the analyzer wafer is about 300 µm.

## 3. Experiment results

In order to verify the applicability of the method for different types of synchrotron radiation light sources, experiments were performed at bending magnet (BM) beamline BL09B and undulator beamline BL16U of Shanghai Synchrotron Radiation Facility (SSRF).

### 3.1. BM beamline

**Figure 3:**
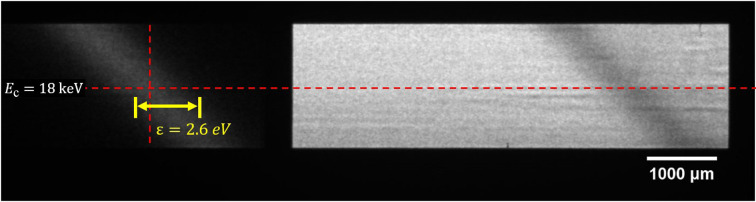
Image of the diffracted (left) and transmitted (right) beam detected by the sCMOS camera at BL09B, in which *E*_c_ is the Bragg energy of the beam and ɛ is the energy bandwidth.

An image of the diffracted (left) and transmitted (right) beam with 4 ms exposure time at BL09B is shown in Fig. 3 ▸. In the experiment, the distance from the light source to the detector was about 40 m and the center energy of the DCM was 18 keV; the corresponding energy resolution of the system was 26 meV. The spot size of the transmitted beam in Fig. 3 ▸ is about 6.2 mm × 1.8 mm, which is primarily determined by the opening size of the slit upstream of the DCM. The integral intensity ratio of the diffracted and transmitted beam is 1:13, which can be adjusted by changing the Bragg angle and thickness of the orthogonal analyzer to ensure sufficient photon flux in the transmitted beam. According to the law of energy conservation, the intensity distributions of the diffracted beam and the dark area in the transmitted beam are completely complementary. Therefore, the stability characteristics of the transmitted beam can be obtained by analyzing the intensity distribution of the diffracted beam. The longitudinal intensity distribution at the center of the diffracted beam is fitted by a Gaussian function, revealing an energy bandwidth of 2.6 eV (FWHM) and a corresponding Darwin width of 16 µrad. The measured energy bandwidth is roughly 13% larger than the theoretical value, which indicates that the analyzer used does not have evident lattice distortions, as can also be seen from the intensity uniformity of the two beams. The intensity distribution of the diffracted beam at different times can be recorded by changing the sampling frequency while keeping the exposure time unchanged. Then the jitter in high-frequency sampling and drift in low-frequency sampling can be calculated according to the peak-position variation in the Gaussian fitting.

**Figure 4:**
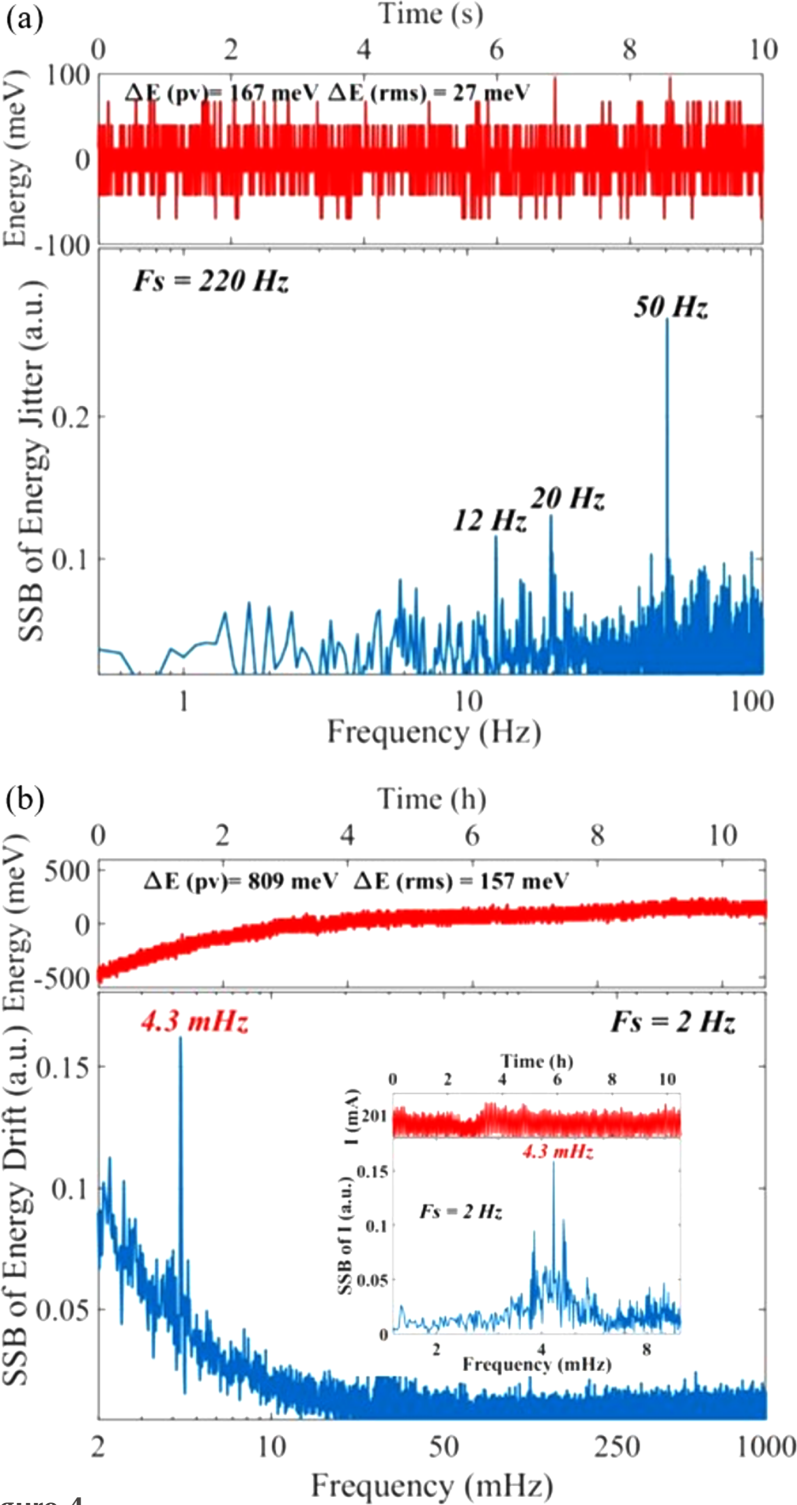
(*a*) Variation of the center energy in 10 s and the corresponding SSB of the energy jitter at BL09B. (*b*) Variation of the center energy over 10 h and the corresponding SSB of the energy drift at BL09B, in which the interior graph represents the variation of electron beam current in the storage ring.

The results of the high-frequency characteristics of the beam energy are shown in Fig. 4 ▸(*a*), in which the sampling frequency is 220 Hz and the sampling time is 10 s. The root-mean-squared (RMS) and peak-to-valley (PV) values of the energy jitter are 27 meV and 167 meV, respectively. The corresponding single-sideband spectrum (SSB) given by fast Fourier transform (FFT) of the energy jitter has three obvious characteristic frequencies, which are 12 Hz, 20 Hz and 50 Hz. The largest characteristic frequency in the SSB is 50 Hz, which is the frequency of alternating current in China. The contribution of the other two characteristic frequencies, 12 Hz and 20 Hz, generated by the vibration of various instruments around the beamline station, is almost equal. The results of the low-frequency characteristics of the energy are shown in Fig. 4 ▸(*b*), in which the sampling frequency is 2 Hz and the sampling time is over 10 h. The RMS and PV values of the energy drift are 157 meV and 809 meV, respectively. The corresponding SSB has a characteristic frequency of around 4.3 mHz which is consistent with the characteristic frequency of electron beam injection in the top-up mode [Fig. 4 ▸(*b*) inset]. Thus, it is evident that the beam injection process has a significant influence on the long-term stability of the electron bunch intensity and the photon beam energy.

### 3.2. Undulator beamline

**Figure 5:**
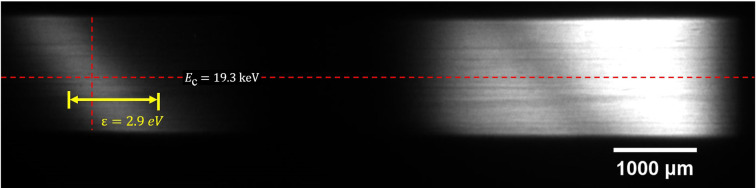
Image of the diffracted (left) and transmitted (right) beam detected by the sCMOS camera at BL16U, in which *E*_c_ is the Bragg energy of the beam and ɛ is the energy bandwidth.

Images of the diffracted (left) and transmitted (right) beam with 1 ms exposure time at BL16U are shown in Fig. 5 ▸. In the experiment, the distance from the light to the detector was about 40 m and the center energy of the DCM was 19.3 keV; then the corresponding energy resolution of the system was 30 meV. The spot size of the transmitted beam in Fig. 5 ▸ is about 3.8 mm × 1.4 mm and the integral intensity ratio of the diffracted and transmitted beam is 1:7. Compared with the BM source, the beam from the undulator source has smaller angular divergence and higher brightness, which means higher requirements for measurement, especially the crystal quality and system resolution. The longitudinal intensity distribution at the center of the diffracted beam is fitted by a Gaussian function, revealing an energy bandwidth of 2.9 eV (FWHM) and a corresponding Darwin bandwidth of 15.5 µrad. The measured Darwin bandwidth and energy bandwidth are roughly 16% larger than the theoretical value.

**Figure 6:**
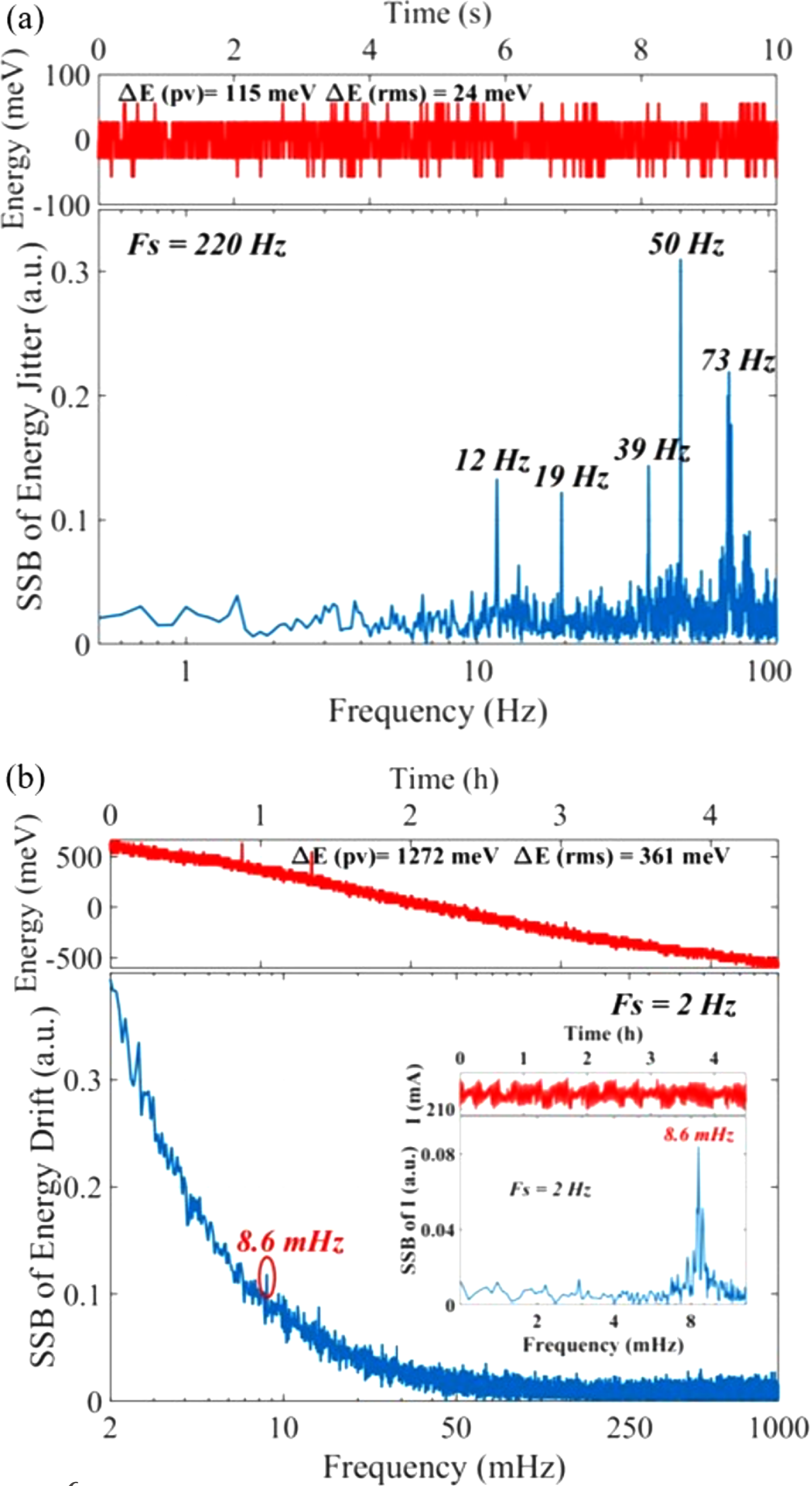
(*a*) Variation of the center energy in 10 s and the corresponding SSB of the energy jitter at BL16U. (*b*) Variation of the center energy over 4 h and the corresponding SSB of the energy drift at BL16U, in which the interior graph represents the variation of electron beam current in the storage ring.

The results of the high-frequency characteristics of the beam energy are shown in Fig. 6 ▸(*a*), in which the sampling frequency is 220 Hz and the sampling time is 10 s. The RMS and PV values of the energy jitter are 24 meV and 115 meV, respectively. The corresponding SSB given by the FFT of the energy jitter has five obvious characteristic frequencies, which are 12 Hz, 19 Hz, 39 Hz, 50 Hz and 73 Hz. The largest characteristic frequency is the same as for BL09B, which is the frequency of the alternating current in China. The other four characteristic frequencies are different from BL09B, generated by the vibration of various instruments around the beamline station. The results of the low-frequency characteristics of the energy are shown in Fig. 6 ▸(*b*), in which the sampling frequency is 2 Hz and the total sampling time is over 10 h. However, the electron beam was lost during the experiment and the effective sampling time was over 4 h. The RMS and PV values of the energy drift are 361 meV and 1272 meV, respectively. The corresponding SSB has a characteristic frequency of around 8.6 mHz which is consistent with the characteristic frequency of electron beam injection in the top-up mode [interior graph in Fig. 6 ▸(*b*)]. The experiment results demonstrate that the energy jitter of the two beamline stations is comparable while the energy drift of the undulator beamline is much larger than that of the BM beamline, which may be due to two reasons: one is that the energy of the photons radiated by the undulator is more sensitive to the stability of the electron orbit; the other is due to the difference in the high-heat-load and cooling systems of the DCMs.

## 4. Conclusion

In conclusion, we have established an *in situ*/*operando* method to monitor SR beam stability with meV-level energy resolution and ms-level time response based on the orthogonal diffraction imaging of a Laue crystal/analyzer, which can decouple the energy/wavelength and Bragg angle of the photon beam using the dispersion effect in the diffraction process. The method was used to measure the energy stability of the photon beam on BL09B and BL16U beamlines of SSRF. The results show that the RMS energy jitter of the two beamlines measured in 10 s is of the order of 10 meV, mainly generated by the disturbance of the power grid and the vibration of various instruments around the beamline; the RMS energy drift on BL09B measured over 10 h and on BL16U over 4 h are of the order of 100 meV and 1000 meV, respectively, which are mainly determined by the beam injection process. It is worth mentioning that the short-term (jitter) and long-term (drift) energy variations of the SR beam at any photon energy can be measured just by adjusting the Bragg angle of the orthogonal analyzer. For light sources with lower emittance, smaller divergence and higher brightness, such as X-ray free-electron lasers and diffraction-limited storage rings, a diffraction plane of higher index with much narrower natural bandwidth could be used to disperse the photon beam and the spatial/spectral resolution could be greatly improved by sampling with a higher-resolution detector.
